# Video Game-Based Interventions for Visual Rehabilitation in Childhood Amblyopia: A Systematic Review and Meta-Analysis

**DOI:** 10.3390/children13020278

**Published:** 2026-02-18

**Authors:** Marina Piñar-Lara, Esteban Obrero-Gaitán, Sara Gómez-Molina, Rafael Lomas-Vega, Héctor García-López, Irene Cortés-Pérez

**Affiliations:** 1 CAIT APROMPSI, C/Clara Campoamor 8, 23470 Cazorla, Spain; mpl00021@red.ujaen.es; 2 Department of Health Sciences, University of Jaén, Campus Las Lagunillas s/n, 23071 Jaén, Spain; rlomas@ujaen.es (R.L.-V.); icortes@ujaen.es (I.C.-P.); 3 Diana del Castillo Physiotherapy Centre, C/Pedro Campos Campiña, 5, 2330 Jaén, Spain; 4 Department of Nursing, Physiotherapy and Medicine, University of Almería, Ctra. Sacramento s/n, La Cañada de San Urbano, 04120 Almería, Spain; hector.garcia@ual.es

**Keywords:** amblyopia, children, meta-analysis, video games, visual acuity, visual rehabilitation, virtual reality-exposure therapy

## Abstract

**Highlights:**

**What are the main findings?**

- Video game-based interventions are effective tools for increasing visual acuity in children with amblyopia.
- The combination of video games and traditional patching therapy demonstrates superior effectiveness compared to patching alone.

**What are the implications of the main findings?**

- Clinicians should consider incorporating video games as an adjuvant to traditional patching.
- Non-immersive systems represent a clinically viable and effective tool, suggesting that specialized or expensive immersive hardware is not strictly necessary to achieve positive visual improvements.

**Abstract:**

Background: Novel approaches such as video games represent a promising tool in increasing visual acuity (VA) in children with amblyopia. The aim was to determine the effectiveness of video game-based interventions (VGBIs) in increasing VA in children with amblyopia. Secondarily, to estimate safety, satisfaction, and compliance with VGBIs. Methods: According to the PRISMA guidelines, a systematic review with meta-analysis (SRMA) was conducted, including studies retrieved from PubMed Medline, SCOPUS, WOS, CINAHL, and PEDro without publication date and language restrictions. We included randomized controlled trials (RCTs) and pilot RCTs, comprising children with amblyopia, that compared the effectiveness of VBGI vs. others in improving VA. Pooled effect was estimated with the Cohen’s standardized mean difference (SMD) and its 95% confidence interval (95%CI). Results: Twenty-one RCTs, providing data from 1515 children, were included. VGBIs are effective (SMD = 0.38; 95%CI 0.08 to 0.68; *p* = 0.013) in increasing VA. Subgroup analyses suggested that non-immersive video games are the most appropriate for improving VA (SMD = 0.35; 95%CI 0.02 to 0.68; *p* = 0.039) and that VGBI is more effective than patching therapy, especially in combination with patching therapy (SMD = 0.63; 95%CI 0.29 to 0.97; *p* < 0.001). Conclusion: This SRMA, including a large number of RCTs to date, demonstrates that VGBI is effective in improving VA in children with amblyopia.

## 1. Introduction

Amblyopia is a neurodevelopmental disorder that significantly affects the visual cortex, defined as the selective suppression of visual information from one or both eyes during the critical period of childhood development [1]. This suppression results in a quantifiable deficit in visual acuity (VA). The global prevalence of amblyopia is estimated at 1.75%, although recent studies highlight a marked geographical variability [2] of 3.67% in Europe and around 0.5% in Africa [3]. Despite regional variations, amblyopia remains a leading cause of pediatric visual disability worldwide [4]. Its incidence in school-aged children, which ranges from 0.1% to 5.5%, highlights the importance of early detection. Amblyopia can affect the performance of daily living activities that require visual information, manifesting in deficiencies in the precision, speed, and variability of movements such as reaching and grasping with the upper extremities, as well as postural stability [5,6]. Studies indicate that children with visual dysfunctions, including amblyopia, may present with comorbidities such as attention deficit hyperactivity disorder [7], dyslexia, and learning difficulties [8]. These alterations can result in decreased academic, physical, and athletic performance, in addition to psychosocial implications such as reduced social contact and stress associated with vision loss [9].

To guarantee the therapy’s success, it is crucial to diagnose it before the age of 7, performing a complete visual examination [10]. The goal of amblyopia treatment is to maximize the connection between the brain and the amblyopic eye to improve VA. The main treatment option is occlusion of the non-amblyopic eye with a patch [11]. Another treatment option is visual penalization with atropine, which has a very similar effect to the patch but a greater degree of acceptance and adherence; however, it can have several systemic adverse effects [12]. Another alternative for penalization is the use of lenses with inaccurate refractive correction, but several studies show that these methods may not be very effective in more severe amblyopia, and they also have a high degree of non-compliance with the therapy [13,14].

On the other hand, the development of alternative therapeutic interventions focused on reducing suppression mechanisms present in the visual cortex has been promoted [15], such as dichoptic training [16]. This type of training, together with the evolution of novel technology, has made virtual reality (VR) and video games and/or serious games emerge as promising tools for the treatment of amblyopia. VR devices can be categorized into immersive VR devices, which provide a complete sense of presence through head-mounted displays (HMDs), and non-immersive systems, where interaction occurs via screens without a full virtual presence [17]. The use of video games with therapeutic purposes using VR devices (video game-based interventions [VGBIs]) aims to induce cortical reorganization and promote the activation of different neuronal connections through the presentation of different and complementary images to each eye [18]. This could translate into improvements in various functional skills, such as VA and stereoacuity [19]. Another great advantage of the use of VR devices is the patient acceptability rate, because children find it striking and attractive.

To date, some studies have assessed the use of VR devices and video games in pediatric ophthalmology therapy. Firstly, a recent systematic review has reported the promising use of VR devices as eye-tracking tools in the field of pediatric ophthalmology [20]. The other three systematic reviews compiled the most recent management options in amblyopia, including a few studies focused on VGBI and VR [21,22,23]. Henández-Rodríguez, CJ, Piñeo, DP et al. (2020), suggested that active vision therapy, including VR and VGBI, could be a promising tool in the recovery of visual acuity in amblyopic children [23]. Alrasheed, SH, and Aldakhil, S (2024) assessed traditional and novel approaches in the management of amblyopia, suggesting that adherence and satisfaction with the therapy can be favored using binocular treatment approaches [21]. Yeritsyan, A et al. (2024) noted that VR aids post-surgical alignment and that dichoptic training involving movies and games has been found to be superior to traditional occlusion therapy for amblyopia recovery [22]. Finally, only one systematic review with meta-analysis (SRMA) directly compared the efficacy of VGBI versus patching therapy in improving VA in children with amblyopia, including 10 trials [24]. Shao, W et al. (2023) [24] concluded that VR interventions significantly improve VA compared to traditional patching, particularly in children under seven years of age and in treatment protocols lasting less than 20 h. Some restrictions shared for all these reviews, such as the application of English language restriction, the inclusion of experimental, observational, and review studies in the same systematic review, and the large number of studies that were not detected in their search strategies, justify the elaboration of a new SRMA. Therefore, the objective of this SRMA was to assess the effectiveness of VGBI in improving VA in children with amblyopia. Secondarily, to assess the VR modality more appropriate to be used and, specifically, to compare the efficacy of VGBI with conventional therapies (patching therapy, spectacles, or sham).

## 2. Materials and Methods

### 2.1. Registration

This SRMA followed the guidelines outlined in the Preferred Reporting Items for Systematic Reviews and Meta-Analyses statement [25] and in the Cochrane Handbook for Systematic Reviews of Interventions [26]. The methodological quality of this SRMA was checked using the AMSTAR 2 checklist [27], and it was prospectively registered in PROSPERO (CRD42025603936).

### 2.2. Literature Search Strategy

Two authors, independently, conducted systematic searches in PubMed Medline, SCOPUS, Web of Science, CINHAL Complete, and PEDro from inception to November 2025, without publication date and language restrictions. Complementarily, authors screened reference lists of previously published studies and gray literature. The search strategy was designed considering the PICOS system [28], as follows: Population (children with amblyopia), Intervention (VGBI), Comparison (others such as patching therapy, spectacles, or sham), Outcome (VA), and Study Design (randomized controlled trials [RCTs] or pilot RCTs). The search strategy was customized for each database according to syntax and index rules, using a combination of Medical Subject Headings (MeSH) and entry terms, combined with the Boolean operators “AND” and “OR”. Table 1 displays the search strategies used in the different databases.

### 2.3. Inclusion Criteria

Two authors, independently, meticulously reviewed all references retrieved by title and abstract. When a record was identified as potentially eligible for inclusion in the SRMA, it was examined in detail by both authors. Any discrepancy was resolved by a third author. The Cohen’s kappa coefficient (κ) was used to assess the agreement of inclusion judgments between these authors [29]. The values of this coefficient were interpreted as degrees of agreement according to Landis and Koch: κ < 0 (non-existent); 0 ≤ κ ≤ 0.2 (non-significant); 0.2 < κ ≤ 0.4 (discrete); 0.4 < κ ≤ 0.6 (moderate); 0.6 < κ ≤ 0.8 (substantial); 0.8 < κ ≤ 1 (excellent) [30].

To be included in this SRMA, each study must meet all inclusion criteria related to the PICOS system. Additionally, the RCTs included must provide quantitative data to assess the variables of interest (VA) in the meta-analysis. On the other hand, studies whose samples mixed healthy and case children, and children with impairments other than amblyopia were excluded.

### 2.4. Data Extraction

Two authors, independently, extracted the following data from the studies included in the review using a specifically designed Microsoft Excel spreadsheet: (1) general study characteristics (authorship, year of publication, country, setting and funding); (2) sample characteristics (total participants, mean age, sex, number of groups, and number of participants in each group); (3) characteristics of VGBI (type of video game, modality of VR, duration and frequency of sessions); (4) characteristics of comparison intervention (type of comparison therapy and duration and frequency of sessions); (5) time of evaluation (immediate assessment or follow-up); and (6) data from the analyzed variables (mean and standard deviation for VA assessment). Additionally, authors registered other data related to satisfaction, compliance, and adverse events of patients in the VGBI group.

### 2.5. Risk of Bias and Evidence Quality Assessment

All these assessments were carried out separately by two authors, and disagreements were solved with a third author. First, the risk of bias was analyzed using the Cochrane Risk of Bias tool [31]. It assesses six domains with seven items: selection bias (sequence generation and concealed allocation), performance bias (blinding of participants and therapists), detection bias (blinding of evaluators), attrition bias (incomplete outcomes data), reporting bias (selective outcomes), and other biases. Each item is scored as “+”, “−”, or “?” for low, high, and unclear risk, respectively.

The evidence quality reported for each outcome was assessed using the GRADE statement and the checklist of Meader (2014) [32,33]. Considering the risk of bias of the RCTs included, inconsistency, imprecision, evidence indirectness, and risk of publication bias, the level of evidence of each finding’s meta-analysis can be quantified as high, moderate, low, or very low.

### 2.6. Statistical Analysis

Comprehensive Meta-Analysis Version 4 (Biostat, Englewood, NY, USA) was used for all analyses [34]. The meta-analysis of an outcome only was performed if at least 2 comparisons per outcome were available. Cohen’s standardized mean difference and 95% confidence interval (95% CI) were calculated in a random-effects model for continuous data [35,36]. The effect size was interpreted as null (SMD 0), low (SMD 0.2), medium (SMD 0.4), and large (SMD > 0.7). Each meta-analysis was graphically displayed in the forest plot [37]. Risk of publication bias was assessed using more than one method: the funnel plot, *p*-value for the Egger test, and the trim-and-fill estimation [38,39,40]. Inconsistency or heterogeneity was calculated with the degree of inconsistency of Higgins (I^2^), the χ-square test, and its *p*-value [41,42]. According to this, heterogeneity can be large (I^2^ > 50%), medium (I^2^ > 50–25%), low (I^2^ 25–5%), or null (I^2^ < 5%) [43].

As additional quantitative analyses, the authors carried out a sensitivity analysis using the leave-one-out method, and two subgroup analyses: (1) to assess the effectiveness of VGBI according to VR modality of video game employed (non-immersive [NIVR] or immersive VR [IVR]); (2) to assess the effectiveness of VGBI according to specific comparisons detected in the RCTs included (VGBI vs. patching therapy, VGBI plus patching vs. patching therapy, VGBI vs. spectacle, and VGBI vs. sham).

### 2.7. Other Analyses

Due to the impossibility of performing more meta-analyses, data related to satisfaction, compliance, and adverse events were assessed using qualitative synthesis.

## 3. Results

### 3.1. Study Selection

**Figure 1:**
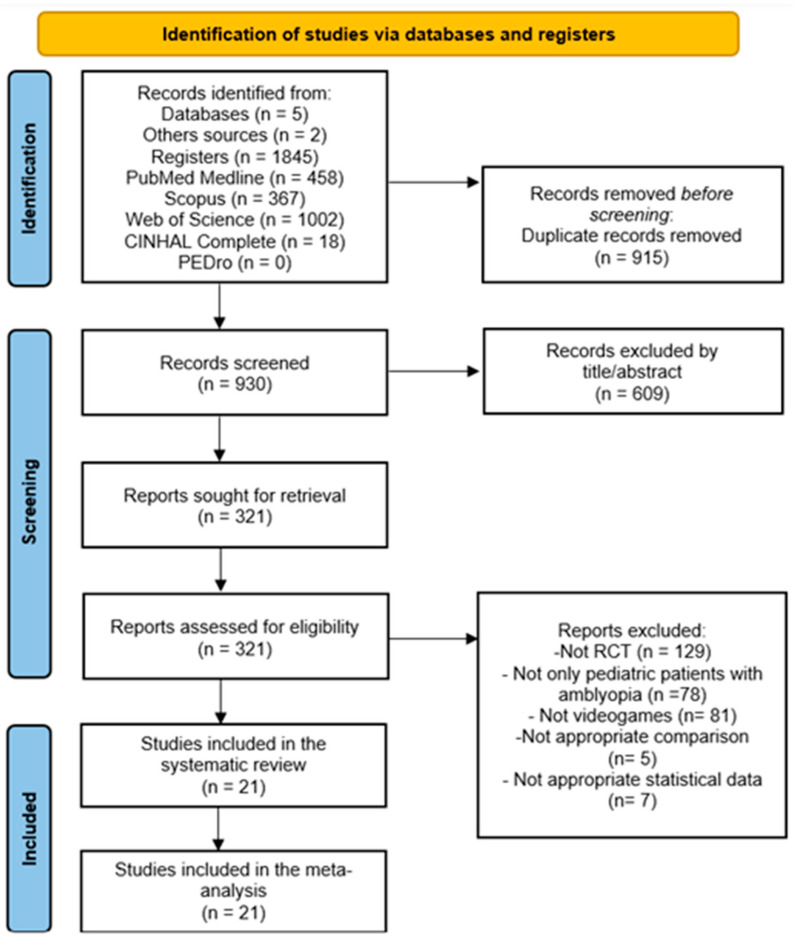
PRISMA flow diagram.

A total of 1847 references were retrieved from databases (n = 1845) and from reference lists of retrieved studies (n = 2). After removing duplicates from the initial screening by title and abstract, 21 RCTs met the inclusion criteria and were included [44,45,46,47,48,49,50,51,52,53,54,55,56,57,58,59,60,61,62,63,64]. The authors demonstrated excellent inter-rater agreement (k = 0.89). Figure 1 shows the PRISMA flow diagram of the study selection process.

### 3.2. Characteristics of the Studies Included in the Systematic Review

The 21 RCTs included, carried out between 2001 and 2024, included 1515 children with amblyopia (mean age of 7.9 ± 2.3 years old), and approximately 46% were boys. The intervention group (VGBI) comprised 750 children, and the comparison group comprised 765 children. In the intervention group, in 17 RCTs, the video games and VR devices used were non-immersive, and only 4 RCTs used immersive VR devices. The duration of the VGBI protocol ranged from 2 to 24 weeks, 1–7 sessions per week, and 20–90 min per session. As a comparison intervention, patching intervention was used in 16 RCTs (of which 3 combined VGBI plus patching therapy), spectacle in 3, and sham in 2. All assessments were carried out just to the end of the intervention (immediate assessment). Finally, 11 RCTs received external funding. More details about the characteristics of these RCTs are summarized in Table 2.

### 3.3. Risk of Bias Assessment

**Figure 2:**
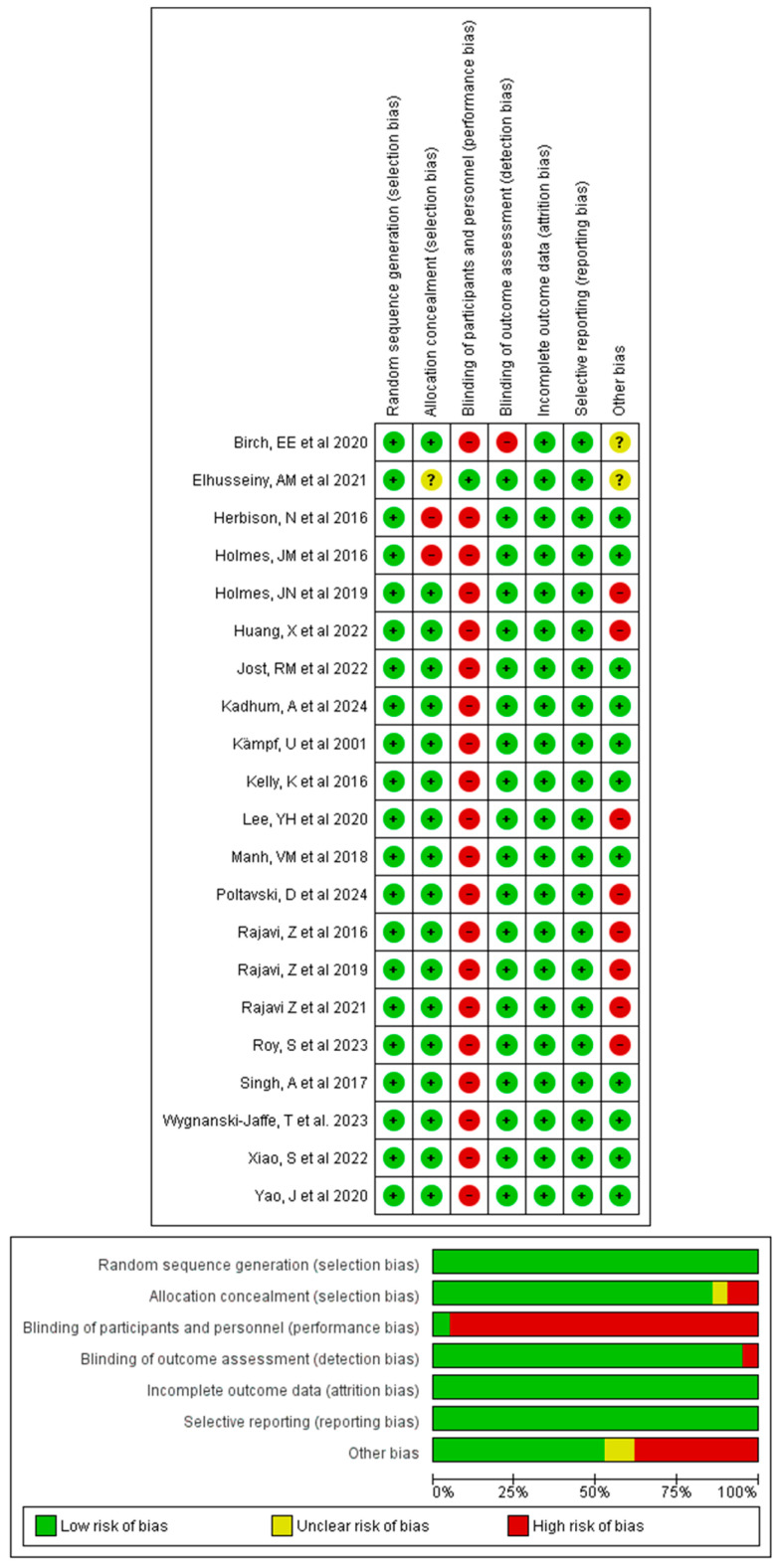
Cochrane Risk of Bias tool assessment [44,45,46,47,48,49,50,51,52,53,54,55,56,57,58,59,60,61,62,63,64].

Random sequence generation, incomplete outcome data, and selective reporting presented low risk in 100% of the studies. Allocation concealment was adequate in 18 studies (85.7%), while two presented a high risk (9.5%) and one presented an uncertain risk (4.8%). Blinding of participants and personnel represented the most affected domain, with high risk in 20 studies (95.2%) due to the impossibility of masking the interventions, and only one study with low risk (4.8%). In contrast, blinding of evaluators was adequate in 20 studies (95.2%), and only one presented a high risk. Finally, the “other sources of bias” domain showed greater variability: 11 studies (52.4%) were classified as low risk, 8 (38.1%) as high risk, and 2 (9.5%) as uncertain. Figure 2 shows the Cochrane Risk of Bias tool assessment.

### 3.4. Quantitative Synthesis: Effect of VGBI on Visual Acuity

**Figure 3:**
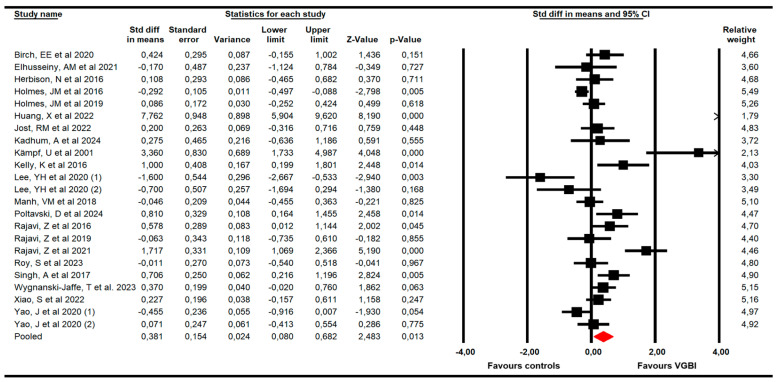
Forest plot of the effect of VGBI on visual acuity in children with amblyopia [44,45,46,47,48,49,50,51,52,53,54,55,56,57,58,59,60,61,62,63,64].

**Figure 4:**
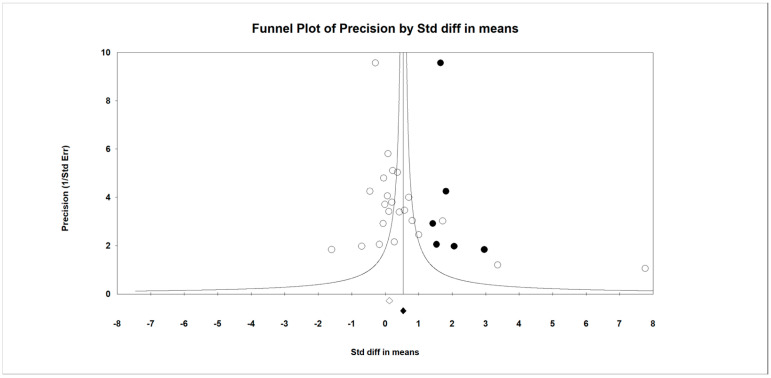
Funnel plot of the effect of VGBI on visual acuity in children with amblyopia (trim-and-fill correction).

Twenty-one RCTs with 23 independent comparisons [44,45,46,47,48,49,50,51,52,53,54,55,56,57,58,59,60,61,62,63,64], providing data from 1550 children with amblyopia (749 receiving VGBI and 801 receiving other interventions), to assess the effectiveness of VGBI on VA. Our meta-analysis showed a low quality of evidence of a medium effect (SMD = 0.38; 95% CI 0.08 to 0.68; *p* = 0.013; I^2^ = 61%; x = 66.1; df = 21; *p* < 0.001) in improving VA favors VGBI (Figure 3). Risk of publication bias was confirmed with the asymmetric funnel plot, Egger test (*p* = 0.004), and trim-and-fill evaluation. Trim-and-fill analysis showed an adjusted pooled effect (adjusted SMD = 0.7; 95% CI 0.37 to 1.1) (Figure 4). This analysis suggests that the risk of publication bias was largely underestimating the true effect of VGBI in improving visual acuity. Finally, sensitivity analysis showed an equal contribution of the studies included in the pooled effect.

#### Subgroup Analyses

**Figure 5:**
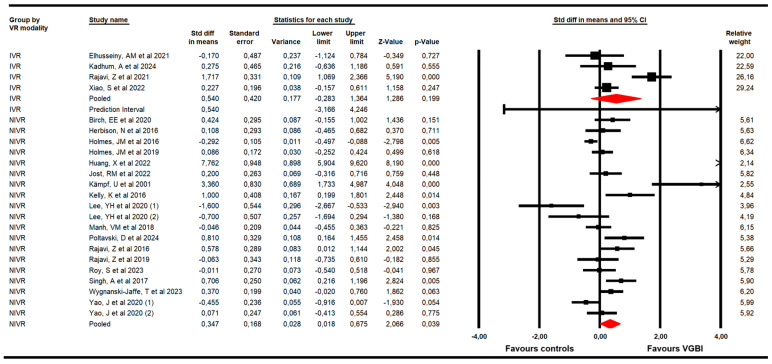
Forest plot of the subgroup analysis according to VR modality [44,45,46,47,48,49,50,51,52,53,54,55,56,57,58,59,60,61,62,63,64].

The first subgroup analysis (Figure 5) showed statistically significant differences in increasing VA after using NIVR (K = 19; SMD = 0.35; 95% CI 0.02 to 0.68; *p* = 0.039; I^2^ = 71.13%; x = 62.4; df = 18; *p* < 0.001) [44,45,47,48,49,50,51,52,53,54,57,59,60,61,62,63,64], but not for IVR devices (K = 4; SMD = 0.54; 95% CI −0.28 to 1.36; *p* = 0.199; I^2^ = 0%; x = 2.9; df = 3; *p* = 0.41).

**Figure 6:**
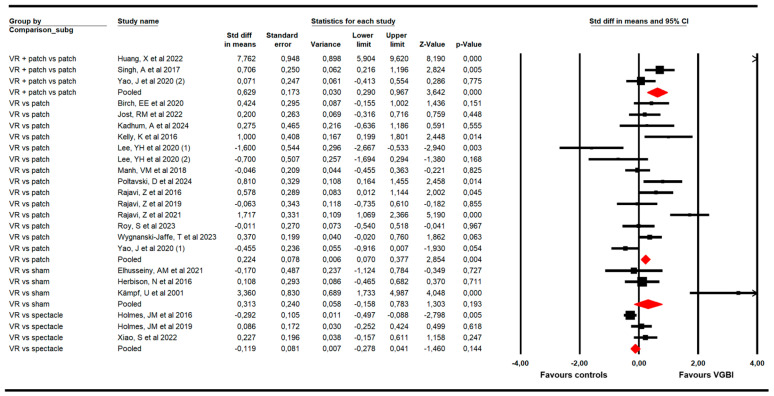
Forest plot of the subgroup analysis according to specific comparisons [44,45,46,47,48,49,50,51,52,53,54,55,56,57,58,59,60,61,62,63,64].

The second subgroup analysis (Figure 6) reported that (1) VGBI was better than patching therapy (K = 14; SMD = 0.22; 95% CI 0.07 to 0.38; *p* = 0.004; I^2^ = 25.4%; x = 17.4; df = 13; *p* = 0.18) [44,45,51,52,53,54,55,58,59,60,61,63]; and that VGBI plus patching treatment showed a major effect compared to only patching treatment (K = 3; SMD = 0.63; 95% CI 0.29 to 0.97; *p* < 0.001; I^2^ = 69.3%; x = 9.17; df = 2; *p* = 0.001) [48,62,64]. Opposite, non-statistically significant differences were found between VGBI and sham [46,49,57] (SMD = 0.31; 95% CI −0.16 to 0.78; *p* = 0.193; I^2^ = 76.62%; x = 12.9; df = 2; *p* < 0.001), and between VGBI and spectacle [47,50,56] (SMD = −0.12; 95% CI −0.28 to 0.04; *p* = 0.144; I^2^ = 61.3%; x = 6.9; df = 2; *p* = 0.03).

### 3.5. Certainty of Evidence 

According to the GRADE assessment and the Meader checklist, overall findings reported low quality of evidence, while findings in the different subgroup analyses ranged from low to very low. Table 3 details the certainty of evidence of the findings of the meta-analyses.

### 3.6. Qualitative Synthesis: Satisfaction, Compliance, and Adverse Events

The qualitative findings reported by RCTs concerning satisfaction, compliance, and adverse events related to the use of VGBI are summarized in Table 4.

Firstly, 3 RCTs [49,56,63] suggested a high level of satisfaction with the therapy, as, overall, 90% of the participating children reported enjoying the therapy and found the systems engaging and easy to use, being recommended for future treatments. Second, 16 RCTs provided data regarding the adherence or compliance of children to VGBI [44,45,46,47,49,50,51,52,53,55,56,58,59,61,63,64], reporting a significantly higher level of adherence in the group receiving VGBI. In the majority of RCTs included, over 60% of participants completed the prescribed treatment [44,45,46,49,51,52,55,56,58,59,63,64]. Finally, 9 RCTs provided relevant data about the safety of VGBI [46,47,49,50,53,56,61,63,64]. Two of these studies reported no adverse effects [46,64], and the remaining seven studies reported a low incidence of mild adverse effects associated with immersive VR, such as double vision, symptoms related to cybersickness, diplopia, blurred vision, or eye discomfort.

## 4. Discussion

Amblyopia presents a significant clinical challenge. Traditionally, passive or conventional therapies, mainly patching and occlusive contact lenses, have demonstrated effectiveness in managing this condition [21,22], with reduced levels of therapy compliance. More recently, “active vision therapy” [23], encompassing methods such as dichoptic therapy, perceptual learning, and particularly vision game-based intervention (VGBI) [24], has emerged as a promising avenue to enhance visual neuroplasticity and improve VA in amblyopic children. The ludic and gamified nature of VGBI is particularly noteworthy, as it has the potential to significantly increase treatment adherence, a common challenge with traditional methods. Given these considerations, this SRMA of RCTs was conducted to systematically elucidate the effectiveness of VGBI in the management of amblyopia in children. We identified 21 RCTs that provide data from 1515 children with amblyopia. Through these RCTs, our meta-analysis elucidates that VGBI can indeed improve VA in amblyopic children.

The primary outcome assessed in this SRMA was VA in amblyopia. Our findings indicate a moderate effect of VGBI in improving VA in children with amblyopia. It is important to note that the presence of publication bias likely led to an underestimation of this effect. Our analysis suggests the true effect could be as much as 84% greater than initially observed. These results align with previous systematic reviews [21,22,23] and meta-analyses [24]. A significant strength of our SRMA is the high level of evidence provided, attributable to the larger number of included studies, the absence of language and publication date restrictions, and the exclusive inclusion of RCTs. This SRMA also yielded other interesting findings from subgroup analyses. Firstly, the video games and virtual VR devices used in the included RCTs were categorized as either non-immersive or immersive. Our SRMA demonstrated that NIVR was the more effective VR modality or VGBI type for increasing VA in amblyopic children. This could be attributed to their simplicity, ease of use, and understanding. This finding represents a novel contribution, as no previous reviews have assessed this specific distinction. Furthermore, when directly comparing the efficacy of VGBI with other interventions included in the RCTs, our results indicate that VGBI was superior to patching therapy in improving VA. This finding directly corroborates the results of Shao et al. (2023) [24], though our SRMA included more RCTs, thereby strengthening the evidence level of these previous observations. Additionally, we uncovered another novel finding: the combination of VGBI and patching therapy appears to be the most effective management strategy for VA, surpassing traditional methods such as patching therapy alone.

A significant challenge in managing amblyopia in children is therapy adherence. While traditional methods, such as patching, have proven effective, they often lead to physical discomfort and social stigma, among others, ultimately reducing compliance [65]. In contrast, video games and VR offer an engaging alternative, supported by recent research highlighting their potential to significantly improve adherence. Studies have shown that gamification, through video games or serious games, by integrating playful elements into visual therapy, increases patient motivation and engagement, transforming the therapeutic routine into an enjoyable and participatory experience [66]. In line with this, our review suggests that VGBI leads to high levels of satisfaction in children, specifically revealing that 90% of children who used these interventions found the therapy enjoyable. Furthermore, the included studies consistently reported higher levels of adherence/compliance in the VGBI group. In most included studies, children completed more prescribed therapy, demonstrating high adherence to VGBI. Beyond the playful component, another crucial element is to know if VGBI produces minimal adverse effects. In this regard, our review demonstrates that the included interventions are safe, showing only minimal adverse effects such as eye discomfort, symptoms of motion cybersickness, or blurred vision.

The effectiveness of VGBI and VR devices in treating amblyopia in children could be attributed to several key factors. Firstly, these video games facilitate the presentation of distinct visual stimuli to each eye, which is crucial for binocular training. Studies have shown that dichoptic visual stimulation (presenting different images to each eye) can induce cortical changes, promoting the recovery of binocular function. VGBI engaging visual stimuli directed at the amblyopic eye stimulates its activity and strengthens these connections. This process gradually restores synaptic balance in the visual cortex, allowing for a more equitable representation of information from both eyes [67]. Secondly, video games and VR foster improved VA through perceptual learning, a process that optimizes the ability to discriminate and recognize visual stimuli through repetitive practice [68]. Research has demonstrated that immediate visual feedback and intrinsic rewards provided in interactive virtual environments accelerate perceptual learning, strengthening neural connections associated with visual processing [69]. VR, by integrating multisensory stimulation such as auditory and haptic feedback, further enhances this learning by increasing patient attention and immersion [70]. This adaptability of VR allows for the customization of visual stimuli, which is crucial given the heterogeneity of amblyopia. Finally, the accessibility of VR systems and video games through portable and home-use devices facilitates remote monitoring of treatment progress, allowing for early interventions [71]. In this sense, it is crucial to increase parents’ involvement in the therapy [72]. Investigations have confirmed that parental supervision and support significantly increase treatment compliance and, consequently, the achieved outcomes.

Although the findings of this SRMA update the scientific evidence about the effectiveness of VGBI in the treatment of amblyopia, some limitations can be considered. Firstly, although the number of studies included in the SRMA is large, specifically, the number of studies in which immersive VR or other specific comparisons have been assessed is low. Second, the presence of potential selection, performance, and detection biases in the studies included may obscure the true effect of VGBI [73,74]. Thirdly, meta-analysis showed a large risk of publication bias that can alter the true effect of VGBI. Trim-and-fill evaluation demonstrated that the risk of publication bias underestimates the true effect. Another limitation is related to the large statistical heterogeneity found in the meta-analyses, related to VGBI protocols, measurement tools, and control comparisons in the studies included. However, sensitivity analysis showed a homogeneous contribution of studies in the pooled effect. Finally, these findings show the effectiveness of VGBI in the short-term (post-intervention assessment), and no follow-up could be assessed because the studies included did not provide data. Future studies must focus on the evaluation of the effectiveness of IVR devices and on the assessment of whether the effect of VGBI is maintained over time. Additionally, future research is needed to determine the optimal parameters for visual stimulation, the duration of treatment, and the frequency of sessions. These studies will guarantee to consolidate these promising findings.

## 5. Conclusions

This systematic review with meta-analysis conclusively demonstrated the effectiveness of vision game-based intervention for improving visual acuity in children with amblyopia. On the one hand, non-immersive virtual reality is the most appropriate virtual reality modality to use it, and specifically, vision game-based intervention was more effective than patching therapy. Our systematic review with meta-analysis reported that the major effectiveness was found when vision game-based intervention was combined with patching therapy. Additionally, children involved in vision game-based intervention reported higher levels of satisfaction and compliance with the prescribed therapy. Finally, vision game-based intervention is safe therapy due to the minimal adverse events reported for amblyopic children.

## Figures and Tables

**Table 1:** Search strategies used in each database.

Databases	Search Strategy
PubMed Medline	(amblyopia[mh] OR amblyopia[tiab] OR lazy eye[mh] OR lazy eye*[tiab] OR developmental amblyopia[mh] OR developmental amblyopia[tiab] OR vision disorders[mh] OR vision disorders[tiab]) AND (virtual reality[mh] OR virtual reality[tiab] OR virtual reality exposure therapy[mh] OR virtual reality exposure therapy [tiab] OR video games[mh] OR video gam*[tiab] OR videogam*[tiab] OR exergaming[mh] OR exergam*[tiab] OR immersive virtual reality[tiab] OR non immersive virtual reality [tiab] OR wii[tiab] OR nintendo[tiab] OR Kinect[tiab] OR virtual environment[tiab] OR simulator[tiab] OR ipad[tiab])
SCOPUS	TITLE-ABS-KEY(“Amblyopia” OR “visual disorders” OR “visual disease”) AND TITLE-ABS-KEY(“virtual reality” OR “virtual reality exposure therapy” OR “wii” OR “kinect” OR “immersive virtual reality” OR “video games” OR “videogame” OR “exergaming” OR “exergame” OR “exergames”)
CINAHL Complete	AB(Amblyopia OR visual disorders OR visual disease) AND AB(virtual reality OR virtual reality exposure therapy OR wii OR kinect OR immersive virtual reality OR video games OR videogames OR exergaming OR exergame OR exergames)
Web of Science	TOPIC(*Amblyopia* OR *visual disorders* OR *visual disease*) AND TOPIC(*virtual reality* OR *virtual reality exposure therapy* OR *wii* OR *kinect* OR *immersive virtual reality* OR *video games* OR *videogames* OR *exergaming* OR *exergame* OR *exergames*)
PEDro	Amblyopia and virtual reality Amblyopia and video games

**Table 2:** Characteristics of the included RCTs in this SRMA.

Study	VGBI Group			Control Group		Visual Acuity (Test)
	Sample Characteristics	VGBI Characteristics		Sample Characteristics	Control Intervention Characteristics	
		System	Duration of VRBT			
Birch, EE et al. 2020 [44] (United States) RCT Funding: Yes, National Eye Institute	23 children 6.7 ± 1.8 years 8B:15G	iPad with a dichoptic game (Dig Rush) with rebalanced contrast, wearing red-green anaglyph glasses	5 weeks per week 60 min per session for 2 weeks	24 children 6.9 ± 1.7 years 8B:16G	Occlusion of non-amblyopic eye for 2 h daily for 2 weeks	e-ETDRS and HOTV
Elhusseiny, AM et al. 2021 [46] (United States) Cross-over double-blind RCT Funding: Yes, Children’s Hospital Ophthalmology Foundation	11 children 7–12 years old (range) 5B:6G	iPhone 6 Plus smartphone with the prototype therapeutic software, and a Zeiss VR One Plus virtual reality headset, which delivered the visual input to each eye dichotically	8 weeks	9 children 9–11 years old (range) 4B:5G	4 weeks of sham treatment followed by 4 weeks of binocular treatment	e-ETDRS
Herbison, N et al. 2016 [49] (United Kingdom) RCT Funding: Welcome Trust	24 children 5.9 ± 1.2 years 13B:11G	I-BiT DVD	1 session per week 30 min session for 6 weeks	25 children 5.6 ± 1.1 years; 12B:13G	Non-I-BiT Game 1 session per week 30 min per session for 6 weeks	Glasgow acuity cards
	26 children 6 ± 1.3 years 9B:17G	I-BiT Game	1 session per week 30 min per session for 6 weeks			
Holmes, JM et al. 2016 [47] (United States) RCT Funding: Yes, National Eye Institute	190 children 8.4 ± 1.8 years 92B:98G	iPad game (Falling Blocks) used red-green anaglyphic glasses	1 daily session 60 min per session for 2 weeks	195 children 8.6 ± 2 years; 106B:89G	Occlusion of non-amblyopic eye for 2 h daily for 16 weeks	e-ETDRS and HOTV
Holmes, JM et al. 2019 [50] (United States) RCT Funding: Yes, National Eye Institute	69 children 9.6 ± 1.6 years 39B:30G	iPad with a dichoptic game (Dig Rush), wearing red-green anaglyph glasses	5 sessions per week 60 min per session for 8 weeks	69 children 9.6 ± 1.5 years 34B:35G	Spectacles (if worn)	e-ETDRS
Huang, X et al. 2022 [48] (China) RCT Funding: NR	18 children; 4–8 years (range)	3D movies with a one-frame delay and occlusion of non-amblyopic eye for a few hours	2–4 sessions per week 45 min per session over 3 months, maximum 40 sessions	20 children; 4–8 years (range)	Occlusion of non-amblyopic eye	Snellen chart
Jost, RM et al. 2022 [45] (United States) RCT Funding: Yes, National Institutes of Health Grant EY022313	28 children 6 ± 1.4 years 13B:15G	Animated films were modified to allow dichoptic presentation on Nintendo 3DS XL	4–5 h weekly for 2 weeks	30 children 6.1 ± 1.5 years 11B:19G	Occlusion of non-amblyopic eye for 2 h daily for 2 weeks	e-ETDRS and HOTV
Kadhum, A et al. 2024 [55] (Netherlands) RCT Funding: Yes, ODAS Stichting	7 children 4–12 years (range) 38% G	Dichoptic action video game using the Oculus Rift VR goggles	1 session per week 60 min per session for 24 weeks	14 children 4–12 years (range) 59% G	Occlusion of non-amblyopic eye for 2 h daily for 24 weeks	The crowded tumbling E chart
Kämpf, U et al. 2001 [57] (Germany) RCT Funding: NR	7 children 6–13 years (range)	Personal computer games that utilize a contrast-modulated sinusoidal grid and full-time occlusion of non-amblyopic eye	5 sessions per week 20 min per session for 2 weeks	7 children 6–13 years (range)	Full-time occlusion of non-amblyopic eye	Landolt Test
Kelly, K et al. 2016 [51] (United States) Crossover RCT Funding: Yes, Thrasher Research Fund, National Eye Institute, and the Retina Foundation of the Southwest	13 children 6.6 ± 1.4 years 10B:3G	iPad with a dichoptic game (Dig Rush) with rebalanced contrast, wearing red-green anaglyph glasses	5 sessions per week 60 min per session for 2 weeks	14 children 6.7 ± 1.5 years 1OB:4G	Occlusion of non-amblyopic eye for 2 h daily for 2 weeks	e-ETDRS and HOTV
Lee, YH et al. 2020 [54] (United States) RCT Funding: No	7 children 8–18 years (range)	Video game played with polarized lenses	20 min per session daily for 8 weeks	10 children; 8–18 years old (range)	Occlusion of non-amblyopic eye for 2 h for 8 weeks	ETDRS and crowding bars
	7 children 8–18 years (range)	video game played with non-amblyopic eye patched	20 min per session daily for 8 weeks			
Manh, VM et al. 2018 [53] (United States) RCT Funding: Yes, National Eye Institute	40 children 14.3 ± 1.1 years 24B:16G	iPad game (Falling Blocks) used red-green anaglyphic glasses	60 min per session daily for 16 weeks	60 children 14.3 ± 1.1 years 34B:26G	Occlusion of non-amblyopic eye for 2 h daily for 16 weeks	e-ETDRS
Poltavski, D et al. 2024 [52] (United States) RCT Funding: NR	21 children 9.42 years; 11B:10G	Video game (Barron Vision) used red-green anaglyphic glasses	5 sessions per week 20 min per session for 12 weeks	19 children 10.4 years; 8B:11G	Occlusion of non-amblyopic eye for 2 to 6 h daily for 12 weeks	ETDRS and HOTV
Rajavi, Z et al. 2016 [60] (Iran) RCT Funding: NR	25 children 6.3 ± 1.9 years 11B:14G	I-BiT Game used red-green anaglyphic glasses	5 sessions per week 20 min per session for 4 weeks	25 children 5.1 ± 1.6 years; 14B:11G	Occlusion of non-amblyopic eye for 1 to 4 h for 4 weeks	Snellen E-chart
Rajavi, Z et al. 2019 [59] (Iran) RCT Funding: NR	17 children 6.5 ± 2 years 9B:8G	I-BiT Game used red-green anaglyphic glasses	5 sessions per week 20 or 30 min per session for 4 weeks	21 children 7.6 ± 1.6 years 7B:14G	Occlusion of non-amblyopic eye for 2 to 4 h daily and Placebo I-BiT Game used red-green anaglyphic glasses 5 weekly 20 or 30 min sessions for 4 weeks	Snellen E-chart
Rajavi, Z et al. 2021 [58] (Iran) RCT Funding: NR	25 children 6.7 ± 1.9 years 14B:11G	I-BiT Game used red-green anaglyphic glasses	1 session per week 60 min per session for 4 weeks	25 children 7.6 ± 1.7 years 11B:14G	Occlusion of non-amblyopic eye for 2 h daily for 4 weeks	Snellen E-chart
Roy, S et al. 2023 [61] (India) RCT Funding: NR	27 children 11 ± 3.2 years 17B:10G	Dichoptic Tetris video game in smartphone using red-green anaglyph glasses	2 h per day for 3 months	28 children 9.9 ± 3 years 18B:10G	Occlusion of non-amblyopic eye for 6 h daily for 3 months	ETDRS
Singh, A et al. 2017 [62] (India) RCT Funding: NR	34 children 9.9 ± 2.2 years 16B:18G	Fast-action video game and occlusion	60 min per day for 4 weeks	34 children 10 ± 2.2 years 18B:16G	Occlusion of non-amblyopic eye for 6 h daily for 4 weeks	Snellen chart and ETDRS
Wygnanski-Jaffe, T et al. 2023 [63] (Israel) RCT Funding: No	51 children 6.6 ± 1.3 years 28B:23G	CureSight System	5 sessions per week 90 min per session for 16 weeks	52 children 6.9 ± 1.4 years 23B:29G	Occlusion of non-amblyopic eye for 2 h daily for 16 weeks	ATS and e-ETDRS
Xiao, S et al. 2022 [56] (United States) RCT Funding: Yes, Luminopia, Inc.	50 children 6.2 ± 1 years 32B:18G	Dichoptic films with rebalanced contrast in smartphones and virtual reality headsets	6 sessions per week 60 min per session for 12 weeks	54 children 6 ± 1.1years 28B:26G	Wearing glasses full-time	ATS-HOTV
Yao, J et al. 2020 [64] (China) RCT Funding: Yes, the Foundation of Shanghai Municipal Health and Family Planning Commission	33 children 3–13 years (range)	Computer binocular game wearing polarized anaglyph glasses	Two sessions of 20 min per day, for 12 weeks	30 children 3–13 years (range)	Occlusion of non-amblyopic eye for 2 to 6 h daily for 12 weeks	e-ETDRS
	22 children 3–13 years (range)	Computer binocular games wearing polarized anaglyph glasses, plus a control condition				

Abbreviations: VGBI, video game-based interventions; VR, virtual reality; RCT, randomized controlled trial; B, boy; G, girl; NR, non-reported; e-ETDRS, electronic-Early Treatment Diabetic Retinopathy Study protocol; ETDRS, Early Treatment Diabetic Retinopathy Study protocol; ATS-HOTV, Amblyopia Treatment Study HOTV protocol.

**Table 3:** Certainty of evidence (GRADE assessment).

Variable		Certainty of Evidence (GRADE Assessment)					
Visual Acuity		Risk of Bias	Inconsistency	Indirect Evidence	Imprecision	Publication Bias	Evidence Strength
Overall effect (VGBI on visual acuity)		Medium	Yes	No	No	Yes	Low
Subgroup 1	NIVR	Medium	Yes	No	No	Yes	Low
	IVR	Medium	No	No	Yes	Yes	Very low
Subgroup 2	VGBI vs. PT	Medium	Low	No	Low	No	Low
	VGBI + PT vs. PT	Medium	Yes	No	Yes	Yes	Very low
	VGBI vs. sham	Medium	Yes	No	Yes	No	Very low
	VGBI vs. spectacle	Medium	Yes	No	Yes	Yes	Very low

Abbreviations: VGBI, video game-based interventions; NIVR, non-immersive virtual reality; IVR, immersive virtual reality; PT, patching therapy.

**Table 4:** Qualitative findings about satisfaction, adherence, and adverse events in VGBI groups of the studies included.

Study	Satisfaction	Adherence/Compliance	Adverse Events
Birch, EE et al. 2020 [44]	NR	Children in the VGBI group completed 103% prescribed treatment time. Similar results were found in the control group (99% prescribed treatment time)	NR
Elhusseiny, AM et al. 2021 [46]	NR	The adherence in the VGBI group was 80%, while in the control group it was 40%, although this difference was not significant (*p* = 0.08)	None of the patients reported adverse events (diplopia, strabismus, motion sickness)
Herbison, N et al. 2016 [49]	More than 90% of patients enjoyed the therapy, and 67% reported that this game was easy to concentrate on	Adherence was excellent due to most patients using the video game for 30 min at each session	In the VGBI group, ten episodes of adverse events were reported (double vision n = 1, flu or cough n = 2, diarrhea and/or vomiting n = 6, other n = 1)
Holmes, JM et al. 2016 [47]	NR	The adherence was minor in the VGBI group (67%) compared to the patching group (92%). Only 22% of all patients in the VGBI group achieve more 75% of prescribed therapy	Sixteen children reported new tropia and/or worsening of a pre-existing deviation in the VGBI group. Diplopia was infrequent, being the most common less than once a week or once a week (only 8% of participants reported diplopia)
Holmes, JM et al. 2019 [50]	NR	The rate of adherence in the VGBI group (meeting more than 75% of prescribed therapy) was 56% throughout 8 weeks of the therapy	Only 5 patients (7%) reported diplopia less than once a week at the end of the therapy
Jost, RM et al. 2022 [45]	NR	Adherence in the VGBI group was 95% of adherence at the end of the treatment	NR
Kadhum, A et al. 2024 [55]	NR	All children who completed the VGBI achieved the required time (100%)	NR
Kelly, K et al. 2016 [51]	NR	100% of children receiving VGBI game completed the prescribed treatment	NR
Manh, VM et al. 2018 [53]	NR	In the VGBI group, only 13% of patients completed more than 75% prescribed treatment	In the VGBI group, 3 patients reported diplopia (n = 1 less than once a week and n = 2 once a day)
Poltavski, D et al. 2024 [52]	NR	The adherence to the VGBI was 85.7% at the end of the therapy	NR
Rajavi, Z et al. 2019 [59]	NR	The overall compliance in the VGBI group was 87.5%	NR
Rajavi, Z et al. 2021 [58]	NR	All children completed the VGBI therapy, showing a compliance of 100%	NR
Roy, S et al. 2023 [61]	NR	The overall compliance in the VGBI group was 55.6%	Headache, blurred vision, or pain discomfort was reported in the VGBI group (46.1% in younger patients and 21.4% in the older age group)
Wygnanski-Jaffe, T et al. 2023 [63]	88% of parents reported satisfaction with the VGBI, reporting that it is easy to use	The mean adherence of the VGBI was 93%	Eleven cases of adverse events were reported (n = 2 headache and n = 9 allergies)
Xiao, S et al. 2022 [56]	Large satisfaction with digital therapy was shown in Net Promoter Score (>65 points), being largely recommended for future cases	Mean adherence was 98.9% at the end of the prescribed treatment	Twelve cases of adverse events were reported (n = 3 new heterotropia, n = 4 headache, n = 1 strain, n = 4 others such as dizziness, eye twitching, facial redness, and an increase in frequency of night terrors)
Yao, J et al. 2020 [64]	NR	The adherence of the VGBI therapy (completing more than 75% of the prescribed therapy) was 15% in game therapy and 27% in combined game and patching therapy	None of the patients reported adverse events

Abbreviations: VGBI, video game-based interventions; NR, non-reported.

## Data Availability

The data presented in this study are available on request from the corresponding author, the data are not publicly available due to privacy.

## Abbreviations

The following abbreviations are used in this manuscript:

95% CI	95% confidence interval
SMD	Standardized mean difference
SRMA	Systematic review and meta-analysis
RCTs	Randomized controlled trials
VA	Visual acuity
VGBIs	Video game-based interventions
VR	Virtual reality
